# Synthesis of Two-Dimensional Sr-Doped LaNiO_3_ Nanosheets with Improved Electrochemical Performance for Energy Storage

**DOI:** 10.3390/nano11010155

**Published:** 2021-01-09

**Authors:** Bin Zhang, Ping Liu, Zijiong Li, Xiaohui Song

**Affiliations:** 1 School of Physics and Microelectronics, Zhengzhou University, Zhengzhou 450001, China; zb1967@zzu.edu.cn; 2 School of Electric and Information Engineering, Zhongyuan University of Technology, Zhengzhou 450007, China; liu_ping1980@126.com; 3 School of Physics & Electronic Engineering, Zhengzhou University of Light Industry, Zhengzhou 450002, China; 4 Institute of Applied Physics, Henan Academy of Sciences, Zhengzhou 450058, China; xhsong@foxmail.com

**Keywords:** bimetal oxide, nanocomposites, supercapacitor, energy storage

## Abstract

Designing a novel, efficient, and cost-effective nanostructure with the advantage of robust morphology and outstanding conductivity is highly promising for the electrode materials of high-performance electrochemical storage device. In this paper, a series of honeycombed perovskite-type Sr-doped LaNiO_3_ nanosheets with abundant porous structure were successfully synthesized by accurately controlling the Sr-doped content. The study showed that the optimal LSNO-0.4 (La_0.6_Sr_0.4_NiO_3-δ_) electrode exhibited excellent electrochemical performance, which showed a high capacity of 115.88 mAh g^−1^ at 0.6 A g^−1^. Furthermore, a hybrid supercapacitor device (LSNO//AC) based on LSNO-0.4 composites and activated carbon (AC) showed a high energy density of 17.94 W h kg^−1^, a high power density of 1600 W kg^−1^, and an outstanding long-term stability with 104.4% capacity retention after 16,000 cycles, showing an excellent electrochemical performance and a promising application as an electrode for energy storage.

## 1. Introduction 

Worsening environmental problems and growing demand for energy sources stimulate the fast development of clean and renewable power sources as well as progressive electrochemical energy carriers. Supercapacitors, as a promising alternative for fast charge and discharge, long cycle life, efficient and environmentally harmless, have been extensively studied for decades [1,2,3,4,5,6]. In recent years, as a derivative of supercapacitor, the hybrid supercapacitor constructed by a battery-type electrode and an Electronic Double Layer Capacitor (EDLC) type electrode improve the energy density of the device while maintaining high power output, as well as the use of safer and cheaper aqueous electrolytes, which is of great significance to the practical application of the new energy storage device and attracts people’s extensive attention [7,8,9,10,11]. As one of the main factors affecting the performance of hybrid supercapacitor, at present, many studies mainly focused on metal oxide structured electrode materials because of its high specific capacitance (capacity) and high energy density, which is several times higher than that of carbon material electrode [12,13,14]. Therefore, it is still very important to find excellent electrode materials for the development and application of new energy storage devices.

Among these metal oxide electrode materials, bimetallic oxide or trimetallic oxide materials attracted much interest and investigation due to their relatively high activities, attractive low-cost advantage, being environmentally friendly, excellent electrochemical performances, and so on [15,16,17,18,19,20]. Perovskite-type metal oxides, as a kind of functional materials, are widely used in solar cell, catalysis for fuel cells, and metal-air batteries because of their defective structures, good thermal stability and excellent oxygen mobility [21,22,23]. Simultaneously, its excellent energy storage characteristics also attracted more and more attention and research in recent years [24,25]. Johnston et al. [26] found the LaMnO_2.91_ materials with more oxygen vacancies displayed a higher specific capacity and energy storage density than LaMnO_3_ electrode materials, indicating that the metal vacancy defect of cation and anion (oxygen vacancy) are beneficial to the storage of ionic charge of electrolyte, thereby enhancing the electrochemical energy storage performance. Liang et al. [27] studied the electrochemical energy storage property of mesoporous LaNiO_3_/NiO nanostructured thin films, and proved that the electrochemical properties of the composites (LaNiO_3_/NiO) are higher than LaNiO_3_ electrodes. However, due to its poor conductivity, the energy storage performance under high power output was still not ideal, which brings a challenge to the practical application. In the study of the ABO_3_ perovskite-type oxide, modification by the A-site doping is one of the effective methods to further improve the electrochemical performance [28,29,30]. The Sr^2+^ (118 pm) has a radius slightly larger than La^3+^ (103.2 pm). Partial replacement of La^3+^ by Sr^2+^ can increase the average ionic radius of A site, resulting in bond angle distortion, and the change of valence state will also produce more oxygen vacancies and other defects. These defects contribute to enhanced conductivity and charge storability. However, to the best of our knowledge, the Sr-doped on the electrochemical energy storage performance of LaNiO_3_ perovskite nanostructure and the study of perovskite oxide materials in electrode materials are studied not enough at present, which is worthy of further investigation for practical application [27,28,31,32,33].

Herein, we present a simple and scalable sol-gel approach coupled with an annealing treatment to successfully and systematically prepare a series of honeycombed perovskite oxide La_1-x_Sr_x_NiO_3-δ_ composites by Sr-doped with different contents. Research results show that the La_1-x_Sr_x_NiO_3-δ_ composite materials have rich porous structure, and exhibit excellent electrochemical energy storage performance. Moreover, the optimal composite ratio of LSNO-0.4 electrode material with excellent electrochemical energy storage performance is very promising to be used as an alternative electrode material for the practical application of electrochemical capacitor. 

## 2. Experimental Section 

### 2.1. Synthesis of Porous La_1-x_Sr_x_NiO_3-δ_ Nanomaterials

All the experimental reagents were analytical grade. A typical synthesis procedure was as follows: 0.9 mM of La(NO_3_)_3_·6H_2_O, 0.1 mM of Sr(NO_3_)_2_, and 1 mM of Ni(NO_3_)_2_·6H_2_O were dissolved in 25 mL of N,N-Dimethylformamide and magnetically stirred for 3 h. And then, 0.45 g of polyvinylpyrrolidone (PVP-K30) was added into the above mixed solution to get a homogeneous solution by magnetically stirring for 2 h. After that, the homogeneous solution was heated by stirring until the gel formed. Then, the obtained gel was transferred to the crucible and placed in a muffle furnace for heating treatment at 650 °C for 3 h at a heating rate of 5 °C min^−1^. The final product that we prepared is denoted as La_0.9_Sr_0.1_NiO_3-δ_ nanomaterials. A series of La_1-x_Sr_x_NiO_3-δ_ nanomaterials were prepared by controlling the molar ratio of La(NO_3_)_3_·6H_2_O and Sr(NO_3_)_2_ addition (x = 0, 0.2, 0.4, and 0.6).

### 2.2. Materials Characterizations

The XRD patterns were performed to examine the crystal structure of the prepared samples using a Bruker D8 Advance diffractometer with Cu Kα radiation (λ = 1.54056 Å). The microstructure and morphology of the La_1-x_Sr_x_NiO_3-δ_ samples were investigated by a field-emission scanning electron microscope (FESEM; Quanta 250 FEG, USA) and transmission electron microscope (TEM; JEOL JEM-2100, Japan). Scanning electron microscope with energy dispersive spectroscopy (SEM-EDS) was used to analyze the types and contents of elements, and EDS mapping was used to identify the distributions of La, Sr, Ni, and O in the micro areas of sample materials. Nitrogen adsorption-desorption isotherms were performed on an ASAP 2020 analyzer (Norcross, GA). Before the measurements, the samples were degassed in vacuum at 150 °C for 6 h. The mass of degassed sample was about 140 mg. Due to the small specific surface area of the sample, the free volume was reduced by adding a filler rod in the sample tube, so as to improve the test accuracy. The equilibration interval was 30 s, and the number of equilibrium points was 50. The specific surface area (S_BET_) measurements were calculated by a Brunauer-Emmett-Teller (BET). Thermogravimetric analysis (TGA) was performed on SDT Q600 under N_2_ from room temperature to 1200 °C at 10 °C min^−1^.

### 2.3. Electrochemical Measurements

The electrochemical performances were investigated using a CHI660E electrochemical workstation (Shanghai Chenhua, China). The working electrode was prepared by mixing the active materials (as-prepared La_1-x_Sr_x_NiO_3-δ_ nanomaterials), conductive carbon black and polytetrafluoroethylene (PTFE) binder with a mass ratio of 8:1:1, and later thoroughly blended together by adding isopropyl alcohol. After forming a uniform mash, the mixture was casted onto nickel foam current collector (0.8 cm^2^) and pressed together. Then, the electrode was dried in a vacuum oven at 80 °C for 12 h. The mass loading of active material was ~1.9 mg. The electrochemical measurements of the working electrodes were examined in a conventional three-electrode system with 6 M KOH aqueous solution as electrolyte, a Pt plate was used as the counter electrode and saturated calomel electrode (SCE) was used as reference electrode. The specific capacity (*C*) was calculated according to the following equation [28,34]:

(1) $$C=\frac{2I\int V\;dt}{3.6mV}$$

where *C* (mAh g^−1^) is the gravimetric specific capacity, *I* (A) is the discharge current, ∫Vdt (Vs) is the integral area under discharge curve, *V* (V) is the voltage after ohmic drop, *m* (g) is the mass of active material, and 3.6 is a constant derived from the conversion of current (A to mA) and time (s to h) in the whole calculation process of the equation.

For the hybrid supercapacitor device (LSNO//AC), the LSNO-0.4 electrode as positive electrode, AC electrode obtained from purchased as negative electrode and a glass fiber membrane as the separator was assembled into an hybrid supercapacitor device, using 6 M KOH as electrolyte. The mass ratio of the LSNO-0.4 electrode to the AC electrode follows Equation (2). The specific capacity (*Sc*), energy density (*E*), and power density (*P*) were calculated according to the following equations [28,34,35]:

(2) $$\frac{m_{+}}{m_{-}}=\frac{Sc_{-}\times \Delta V_{-}}{Sc_{+}\times \Delta V_{+}}$$

(3) $$Sc=\frac{2I\int V\;dt}{3.6MV}$$

(4) $$E=\frac{I\int V\;dt}{3.6M}$$

(5) $$P=\frac{3600E}{t}$$

where *Sc* (mAh g^−1^) is the specific capacity, *I* (A) is the discharge current, ∫*Vdt* (Vs) is the integral area under discharge curve, *V* (V) is the voltage after ohmic drop, *M* (g) is the mass of active material.

## 3. Results and Discussion

**Figure 1:**
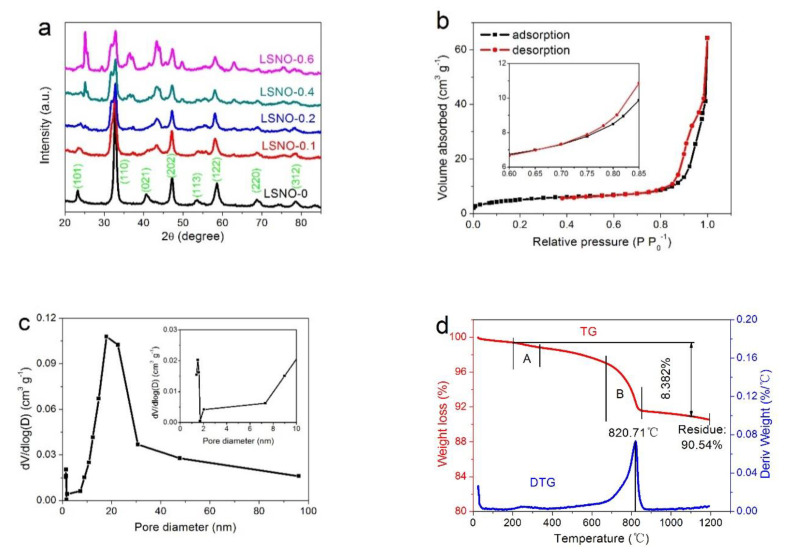
(**a**) XRD patterns of LSNO-x samples; (**b**) N_2_ adsorption-desorption isotherms and (**c**) pore size distributions of the LSNO-0.4 sample, and (**d**) TG and DTG curves of the LSNO-0.4 sample.

It is well known that the different components varying in composite ratio have an influence on the crystal structure and crystal growth of the composite. Therefore, the crystal structure information of the prepared LSNO-x samples are characterized by X-ray diffraction (XRD) patterns in 2θ ranging from 20° to 85°, as illustrated in Figure 1a. From the XRD patterns, we can observe that all LSNO-x samples exhibit almost the same single phase, corresponding to the hexagonal perovskite structure of LaNiO_3_ materials (JCPDS No: 34-1028). These diffraction peaks located at 2θ = 23.3°, 32.9°, 40.7°, 47.4°, 53.6°, 58.7°, 68.9°, and 78.7°, and are indexed to the (101), (110), (021), (202), (113), (122), (220), and (312) planes, respectively. In addition, we can find that the intensity of the main diffraction peaks of the composite decreases obviously with the increase of Sr content (from x = 0 to x = 0.4), such as (110), (202), (122) lattice planes, etc. This result shows that LSNO-0.4 material possesses a lower crystallinity. Moreover, the LaNiO_3_ (ABO_3_) materials that were Sr-doped caused La element to be partially substituted by Sr element, and because the radius of Sr^2+^ (118 pm) ion was slightly larger than La^3+^ (103.2 pm), resulted in the average ionic radius of A site increasing. Therefore, we can find that a small number of diffraction peaks disappeared, shifted, and added with the increase of Sr content. For example, the new peak appears located at around 2θ = 37° should be mainly attributed to the generation of a small amount of by-product NiO materials. Simultaneously, the changes of ionic radius and valence in A-site element led to the emergence of more bond angle distortion and other defects, and thus producing a certain distortion in the lattice of the composite material [28,36]. Meanwhile, the introduction of vacancy defects into the metal oxide structure was also beneficial to improve the electrochemical properties of the material [37,38,39].

For an electrochemical energy storage device, the morphology, microstructure, and porosity have an important effect on the electrode materials, and a higher specific surface area can provide more redox sites and ion storage sites for the increase in capacitance (capacity). Therefore, the different porous properties of the LSNO-x samples are further investigated by N_2_ adsorption-desorption measurements. As shown in Figure 1b, the LSNO-0.4 sample shows a typical type-IV isotherm with a hysteresis loop at a P P_0_^−1^ of about 0.67 (inset in Figure 1b), representing the presence of a large number of mesoporous structures. Detection results (Figure S1 and Table S1) indicate that the LSNO-0.4 sample exhibits a larger BET specific surface area (48 m^2^ g^−1^) and micropore area (16 m^2^ g^−1^) than other LSNO-x samples. In addition, we can observe that the LSNO-0.4 sample had a wide pore size distribution from micropores to macropores, demonstrating an excellent porous nanostructure (Figure 1c). The existence of micro- and mesoporous improved the specific surface area of electrode materials, which provided more reaction sites and enhanced the charge storage capacity. In addition, the mesopores connected with macropores were very conducive to the buffer and diffusion of electrolyte, and reduced the volume change during the charge-discharge cycling, improving the cycle stability. Therefore, the hierarchical porosity composed of micro-, meso-, and macropores was very helpful to improve the electrochemical storage performance of electrode materials. By calculation, we can obtain that the micropores volume in LSNO-0.4 samples was about 15.2% of the total pore volume, which was far higher than other LSNO-x samples (Table S1).

The TG curve of LSNO-0.4 sample shows that the fast weight loss mainly located in phase A and phase B (Figure 1d), corresponding to the evaporation and escape of a small amount of organic polymer and the pyrolysis of LSNO-0.4 materials, respectively. It can be found that the residue weight of the LSNO-0.4 sample was 90.54% after 1200 °C, and compared with the TG curves of other samples (Figure S2), we can directly see that with the increase of Sr-doped content of composite material, the residues weight became smaller after heating to 1200 °C (LSNO-0 (95.46%) < LSNO-0.1 (94.70%) < LSNO-0.2 (93.80%) < LSNO-0.4 (90.54%) < LSNO-0.6 (87.38%)), which indicated that more O elements were involved in the reaction and escaped as gas.

**Figure 2:**
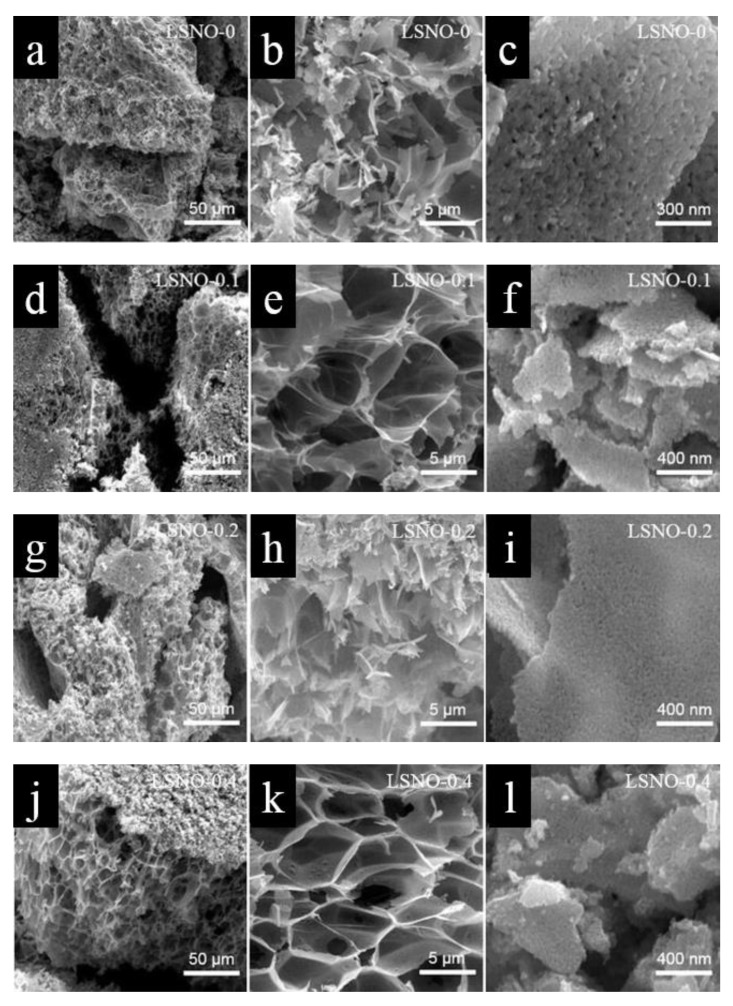

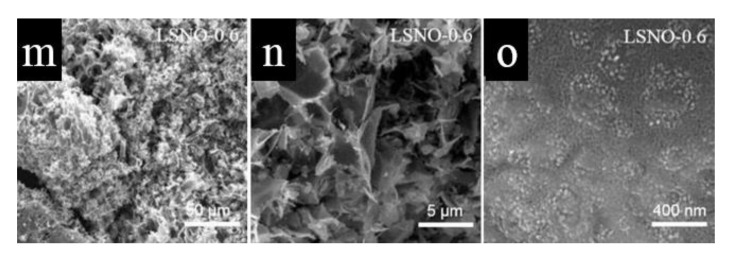
SEM images of LSNOs sample. (**a**–**c**) LSNO-0; (**d**–**f**) LSNO-0.1; (**g**–**i**) LSNO-0.2; (**j**–**l**) LSNO-0.4; (**m**–**o**) LSNO-0.6.

**Figure 3:**
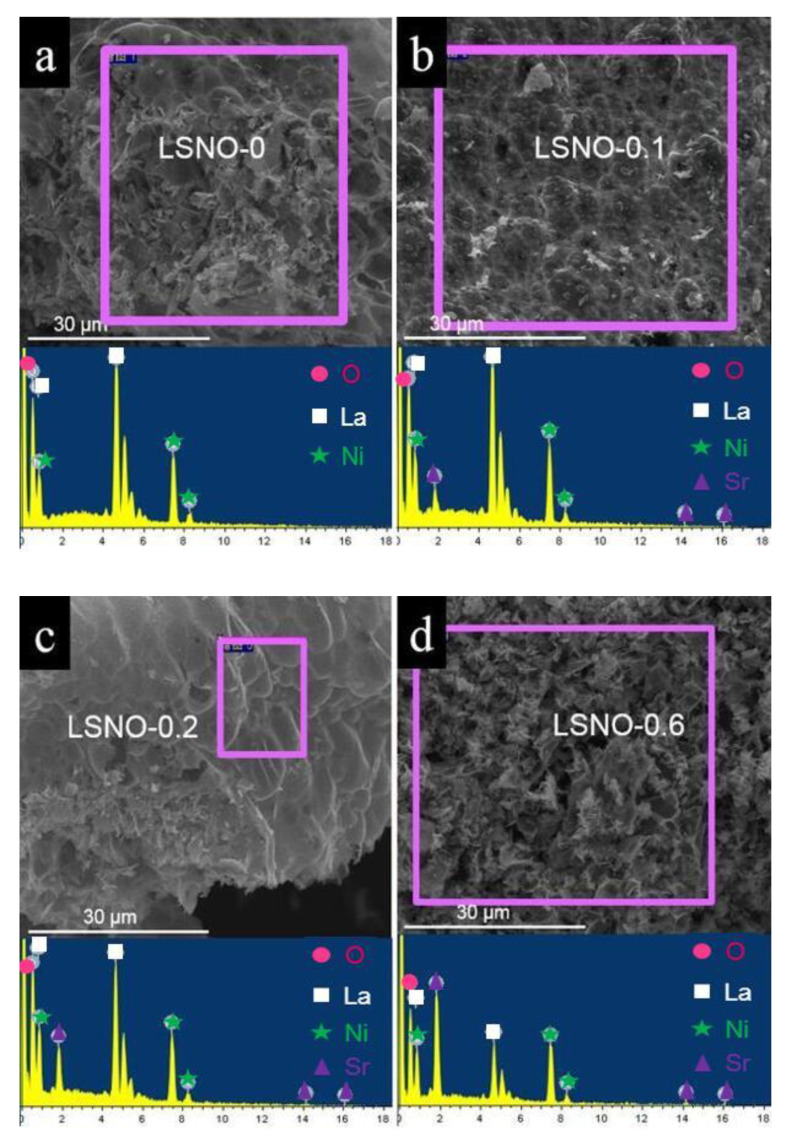

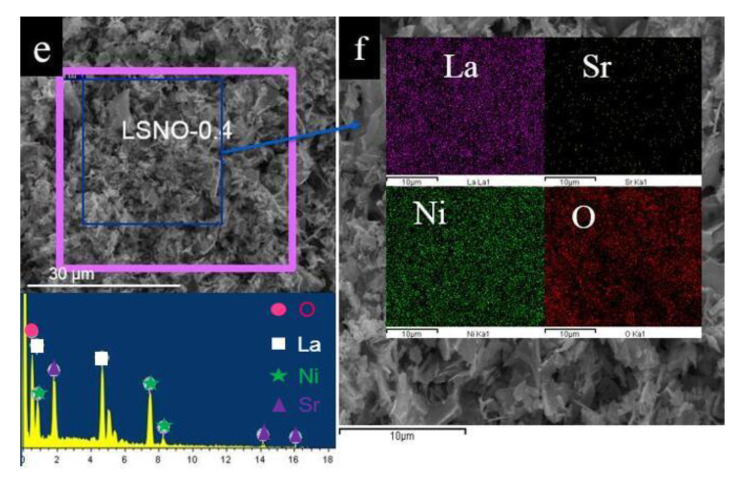
(**a**–**e**) The SEM images with the corresponding energy dispersive spectroscopy (EDS) for LSNOs samples, and (**f**) elemental mapping images of LSNO-0.4 sample.

The microscopic morphology of all prepared LSNOs were characterized by scanning electron microscopy (SEM). As shown in Figure 2j–l, we can observe that the LSNO-0.4 sample possessed a honeycomb nanostructure with rich mesoporous and macroporous, which looked like a large number of honeycombs that are made of nanosheets linked together. In addition, we can find that the surface and fracture surface morphology of materials had great differences, in which the surface was the broken block morphology with a large number of mesoporous structures (Figure 2l), and the fracture surface was the macroporous morphology composed of nanosheets with a honeycomb morphology structure (Figure 2k). Simultaneously, it can also be found that the material surface covered with a massive mesoporous structure is very thin (Figure 2k), which can clearly observe the macroporous structure beneath the thin layer. Compared to the composites obtained with other Sr-doped contents, we found that with the increase of Sr doping content, the composite had a thinner macroporous structure, a mesoporous structure with smaller pore size, and a richer pore structure (Figure 2a–l). However, when the amount of Sr-doped is too high (LSNO-0.6), we could observe that a large number of pore structures collapsed or were destroyed (Figure 2m–o). This result shows that the amount of Sr dopant has a great influence on the morphology and crystal growth of the composite, and the LSNO-0.4 sample has the optimum pore structure and morphology suitable for the storage of energy materials. To further confirm the elemental composition of the prepared LSNO-x composites, energy dispersive spectroscopy (EDS) was also performed. As illustrated in Figure 3a–e, we can clearly see that the Sr elements were detected in the EDS spectra of all LSNO-x (x = 0.1, 0.2, 0.4, 0.6) composites, which proved the existence of Sr in LSNO-x composite material. The elemental mapping images of LSNO-0.4 samples indicated that all the elements can be well dispersed in the composite (Figure 3f), and it also showed that the Sr elements were well doped into the LaNiO_3_ material.

**Figure 4:**
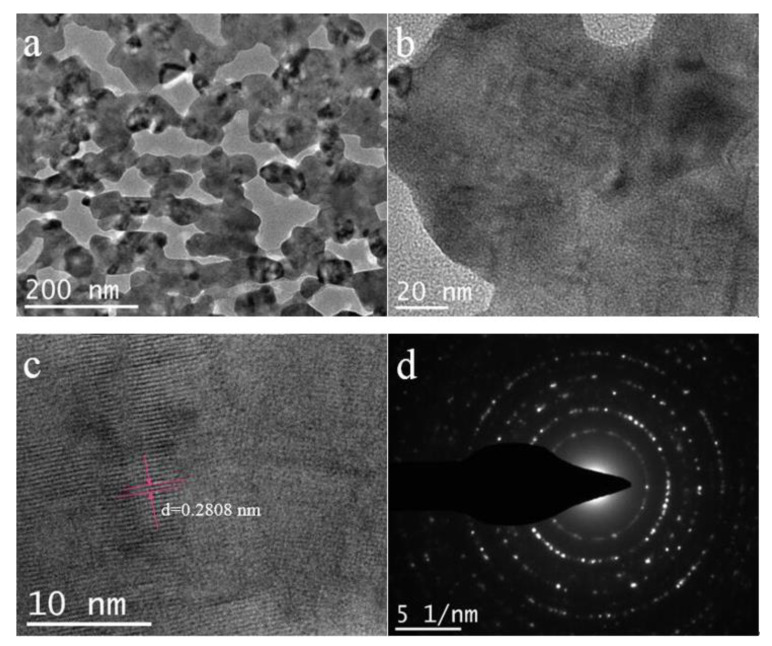
(**a**,**b**) TEM, (**c**) High resolution TEM (HRTEM) and (**d**) Selected area electron diffraction (SAED) images of LSNO-0.4 sample.

In Figure 4a, we can observe that the LSNO-0.4 materials presented a well distributed mesoporous structure, which was mainly produced by gas escaping from the material during calcination. The rich porous nanostructure would enhance the electrochemical performance of the materials as an electrode for energy storage device. High-magnification TEM image shows that the LSNO-0.4 sample presented a relatively transparent structure, which also means very thin nanosheets structure (Figure 4b). Furthermore, the lattice fringes with a lattice distance of about 0.2808 nm (Figure 4c) was slightly larger than that of the LaNiO_3_ sample (0.2735 nm) (Figure S3), which was mainly attributed to the effect of Sr doping (Sr^2+^ (118 pm) > La^3+^ (103.2 pm)). Figure 4d displays the selected area electron diffraction (SAED) image of the LSNO-0.4 sample. Well-defined rings demonstrated the LSNO-0.4 materials possessed a polycrystalline phase, as well as a good crystallinity, which was consistent with the XRD analysis.

**Figure 5:**
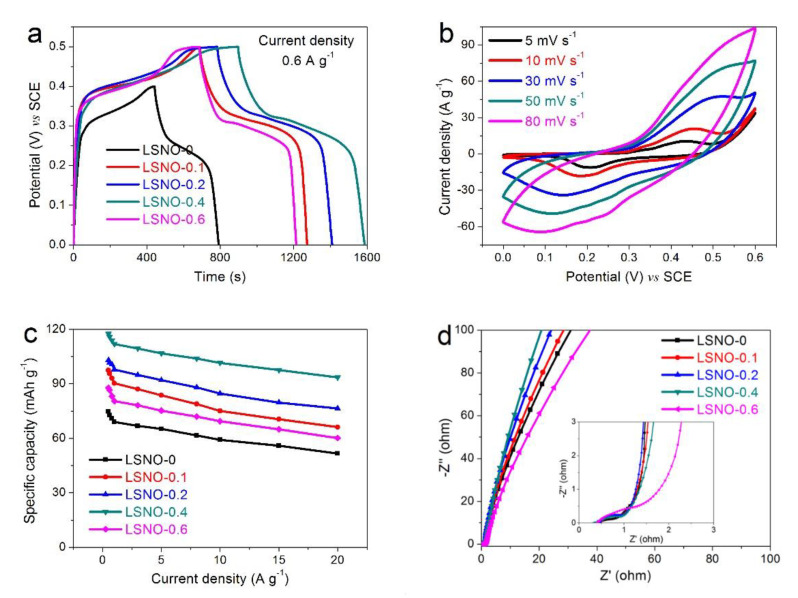
The electrochemical performances of all electrodes in a three-electrode system with 6 KOH aqueous solution as electrolyte: (**a**) galvanostatic charge discharge (GCD) curves of the LSNO-0, LSNO-0.1, LSNO-0.2, LSNO-0.4, and LSNO-0.6 samples at current density of 0.6 A g^−1^; (**b**) cyclic voltammetry (CV) curves of LSNO-0.4 electrode at different scan rates ranging from 5 mV s^−1^ to 80 mV s^−1^; (**c**) Specific capacity vs. Current density, (**d**) Nyquist plots.

The effect of Sr-doped content on the electrochemical properties of LaNiO_3_ electrode materials was evaluated by cyclic voltammetry (CV), galvanostatic charge discharge (GCD), and electrochemical impedance spectroscopy (EIS). As illustrated in Figure 5a, we can clearly see that the specific capacity of the Sr-doped composite was obviously improved (LSNO-0 (72.72 mAh g^−1^) < LSNO-0.6 (86.56 mAh g^−1^) < LSNO-0.1 (95.72 mAh g^−1^) < LSNO-0.2 (101.16 mAh g^−1^) < LSNO-0.4 (115.88 mAh g^−1^). Moreover, with the increase of Sr-doped molar ratio to 40%, the LSNO-0.4 had the largest specific capacity. But the capacity decreased when the Sr-doped molar ratio was further increased to 60%. This result can also be proved by CV curves. As shown in Figure S4, we can easily see that the position of the redox peak of the composite electrode changed significantly, compared to the LSNO-0 electrode, and the potential window also increased. Additionally, from the CV curves of different electrodes at different scan rates, we can find that the oxidation and reduction peaks shifted to both ends, which may be attributed to the increase of the internal diffusion resistance in the electrode with the scan rate increasing (Figure S4 and Figure 5b). The results show that the capacity of the composite electrode material is mainly produced by oxidation-reduction reaction. In addition, a very small IR-drop can be observed in the discharge curve, meaning a very low internal resistance of all electrodes (Figure 5a). Furthermore, the specific capacity of LSNO-0.4 electrode was still maintained 93.61 mAh g^−1^ when the discharge current density increased to 20 A g^−1^, indicating an excellent rate capability (Figure 5c). 

The Nyquist plots of all electrodes are displayed in Figure 5d. All curves present a quasi-semicircle in the high-frequency region and approximately straight lines with a high slope in the low-frequency region, indicating a relatively ideal capacitor behavior. What is more, we can observe that the LSNO-0.4 electrode presented the smallest resistance of electrolyte (Rs) (insert in Figure 5d), which obtains from the intercept on the real axis in the high-frequency region. The semicircle in the middle-high frequency region represents the charge-transfer resistance (*R_ct_*), from which we can see a smaller *R_ct_* in all electrodes, especially for LSNO-0.1, LSNO-0.2, and LSNO-0.4 electrodes (the insert in Figure 5d), and in the low-frequency region, a higher slop of inclined line indicates a smaller Warburg impedance (*Wo*) in LSNO-0.4 electrode, meaning a better ion diffusion efficiency, which was consistent with the lower IR-drop in Figure 5a [13,40].

Compared with other ABO_3_-based electrode materials (Table S2), the enhancement of electrochemical performance may be attributed to the following factors: (1) the porous and honeycombed nanosheet structures can increase the specific surface area of electrode materials, which was not only beneficial to the sufficient and effective contact between the electrode active material and the electrolyte, but also beneficial to the increase of the reaction site for the redox reaction. Therefore, the electrode material had fast ion mobility that was regarded as favoring the improvement of capacity; (2) vacancy defects can be greatly increased in Sr-doped LaNiO_3_ materials, and the more vacancy defects created will be more helpful to contribute to the conductivity and improve the charge storability. However, excessive Sr doping will cause lattice distortion and destroy the ABO_3_ perovskite structure [28]. (3) Peroviske-type ABO_3_ materials exhibited being highly thermal and mechanically stable, which is very good for electron/ion transportation and long cycling life.

**Figure 6:**
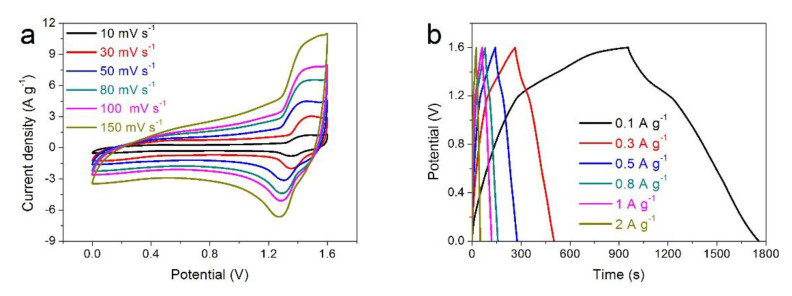

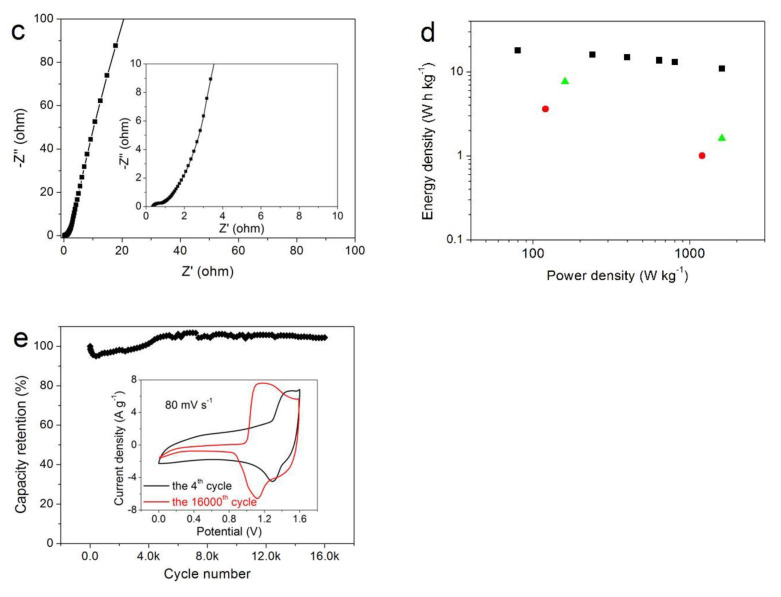
The electrochemical performances of LSNO //activated carbon (AC) hybrid supercapacitor device with 6 KOH aqueous solution as electrolyte: (**a**) CV curves; (**b**) GCD curves; (**c**) Nyquist plots; (**d**) Ragone plot ((circle) [41], (triangle) [42], (square) This work), and (**e**) Cycling performance of the LSNO//AC hybrid supercapacitor device.

To further investigate the practicability of LSNO-0.4 electrode, a simple hybrid supercapacitor device (LSNO//AC) was assembled by using the LSNO-0.4 electrode as the cathode, the activated carbon (AC) electrode as the anode (Figure S5), and a cellulose paper as the separator in 6 M KOH electrolyte. The CV curves of the LSNO//AC hybrid supercapacitor device were collected at scan rate of 50 mV s^−1^ in different potential windows ranging from 0–1 to 0–1.7 V (Figure S6). From these curves, we can obtain that the potential window from 0 to 1.6 V was the optimum choice, which is not only can effectively incorporate the capacity produced by redox reaction, but also remove the irreversible reactions (such as: the CV curve of 0-1.7 V is slightly deformed and raised at the high potential end). The CV curves of the optimized LSNO//AC hybrid supercapacitor device at different scan rates ranging from 10 to 150 mV s^−1^ are displayed in Figure 6a. We can observe that all CV curves remained in a similar shape even at large scan rates, indicating an excellent rate capacity. The presence of an obvious redox peak on the CV curve indicated the existence of Faraday capacity. In addition, with the increase of scanning rate, the position of the oxidation peak and the reduction peak moved slightly to both ends, indicating that the redox reaction of the LSNO//AC hybrid supercapacitor device had excellent reversibility. The nonlinear GCD curves shown in Figure 6b further prove the generation of Faraday’s behavior of the device. From the Nyquist plots in Figure 6c, we can obtain that the hybrid device exhibited a low resistance of electrolyte (~0.35 Ω), charge-transfer resistance (~0.8 Ω), and Warburg impedance (*Wo*), showing a good conductivity and ion diffusion efficiency. In addition, the hybrid supercapacitor device possessed a high energy density of 17.94 W h kg^−1^ at a power density of 80 W kg^−1^, and still maintained a high energy density of 10.89 W h kg^−1^ at a power density of 1600 W kg^−1^ (Figure 6d), which was higher than those previously reported energy storage device with similar electrode material [41,42]. Figure 6e shows the cycling performance of the LSNO//AC hybrid device at a scan rate of 80 mV s^−1^. We can observe that the capacity decrease during the first 600 cycles, which is mainly due to a small amount of unstable structure and irreversible reaction in the composite. After that, with the increase of the cycle numbers, the capacity gradually increased, and reached the maximum value at about 7000 cycles, which was about 106.8% of the initial capacitance. This is mainly due to the continuous and complete penetration of the electrolyte, which promoted the full activation of the internal reaction sites, thus improving the capacity. This can be seen from the increase in the area enclosed by the curve of the redox peak in the insert of Figure 6e. Then, with the further increase of the cycle numbers, the capacity decreased slightly, and the overall trend tended to be stable. And after 16,000 cycles, the hybrid supercapacitor device still retained 104.4% of the initial capacity, showing excellent cycle stability, which was mainly attributed to the stable nanostructure of ABO_3_ perovskite oxide. 

## 4. Conclusions

Honeycombed perovskite-type Sr-doped LaNiO_3_ nanosheets with rich porous structure were successfully synthesized by accurately controlling the Sr-doped content via a simple sol-gel method. The optimal LSNO-0.4 composite electrode exhibited the best electrochemical performance with a high specific capacity of 115.88 mAh g^−1^ at 0.6 A g^−1^. When applied in a LSNO//AC hybrid supercapacitor device, the device delivered a high energy density of 17.94 W h kg^−1^, a high power density of 1600 W kg^−1^, as well as an outstanding cycling performance with 104.4% of the initial capacity after 16,000 cycles, indicating an excellent electrochemical performance. All these results show that the LSNO perovskite oxide with an excellent performance has a great potential as a substitute material for the application of electrochemical energy storage device.

## Data Availability

Data available in a publicly accessible repository.

## Supplementary Materials

The following are available online at https://www.mdpi.com/2079-4991/11/1/155/s1, Figure S1: The comparison of (a) N2 adsorption-desorption isotherms and (b) pore size distributions of LSNO-x samples at 77 K. Figure S2: The comparison of TG curves of other LSNO-x samples. Figure S3: High-resolution TEM (HRTEM) of LaNiO_3_ sample. Figure S4: (**a**–**d**) Cyclic voltammetry (CV) curves of the LSNO-0, LSNO-0.1, LSNO-0.2 and LSNO-0.6 electrodes at different scan rates ranging from 5 to 50 mV s^−1^. Figure S5: (**a**) CV curves, (**b**) GCD curves, (**c**) Nyqusit plots and (**d**) Cycling performance of the prepared activated carbon (AC) electrode in 6 M KOH electrolyte. Figure S6: CV curves at different potential window ranging from 0–1 V to 0–1.7 V at a scan rate of 30 mV s^−1^. Table S1: Textural parameters of the LSNO-x samples. Table S2: Comparison of capacitive performance based on ABO_3_-based electrode materials in the literatures.
