# Supplementary material for: Synthesis of Two-Dimensional Sr-Doped LaNiO3 Nanosheets with Improved Electrochemical Performance for Energy Storage
Source: Nanomaterials (Basel). 2021 Jan 9;11(1):155. doi: 10.3390/nano11010155 (PMC7827135; doi:10.3390/nano11010155)
Supplement: Supplementary file 1 [file nanomaterials-11-00155-s001.pdf]

nanomaterials

Supplementary Information

Synthesis of Two-Dimensional Sr-Doped $LaNiO_3$ Nanosheets with Improved Electrochemical Performance for Energy Storage

Bin Zhang [1], Ping Liu [2], Zijiong Li [3,*] and Xiaohui Song [4]

1 School of Physics and Microelectronics, Zhengzhou University, Zhengzhou 450001, China; zb1967@zzu.edu.cn
2 School of Electric and Information Engineering, Zhongyuan University of Technology, Zhengzhou 450007, China; liu_ping1980@126.com
3 School of Physics & Electronic Engineering, Zhengzhou University of Light Industry, Zhengzhou 450002, China
4 Institute of Applied Physics, Henan Academy of Sciences, Zhengzhou 450058, China; xhsong@foxmail.com
* Correspondence: zijiongli@zzuli.edu.cn

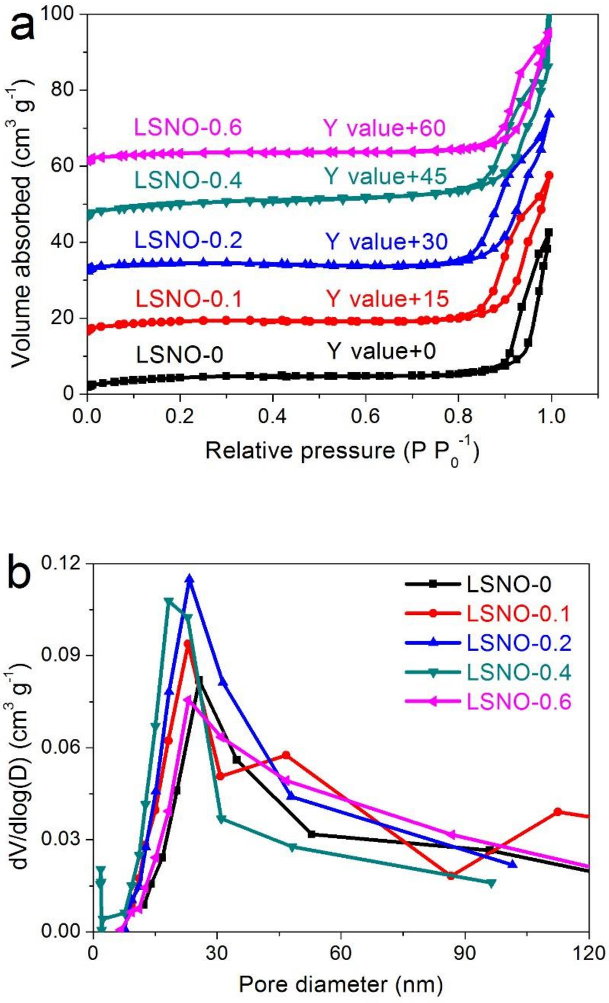

Figure S1. The comparison of (**a**) N_2 adsorption-desorption isotherms and (**b**) pore size distributions of LSNO-x samples at 77 K.

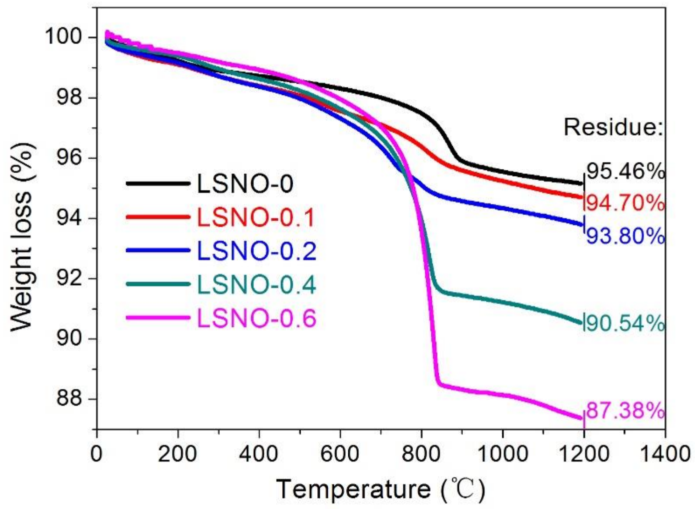

Figure S2. The comparison of TG curves of other LSNO-x samples.

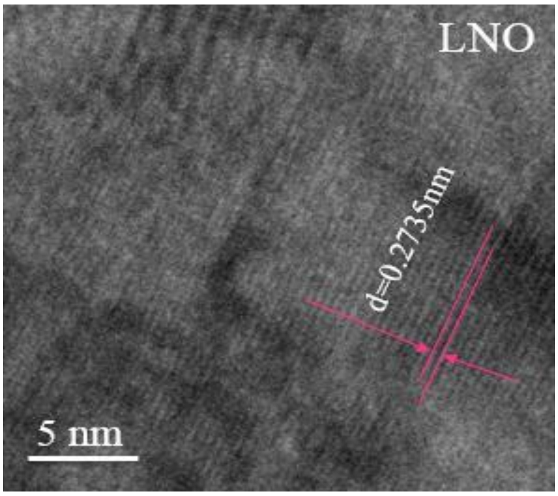

Figure S3. High-resolution TEM (HRTEM) of $LaNiO_3$ sample.

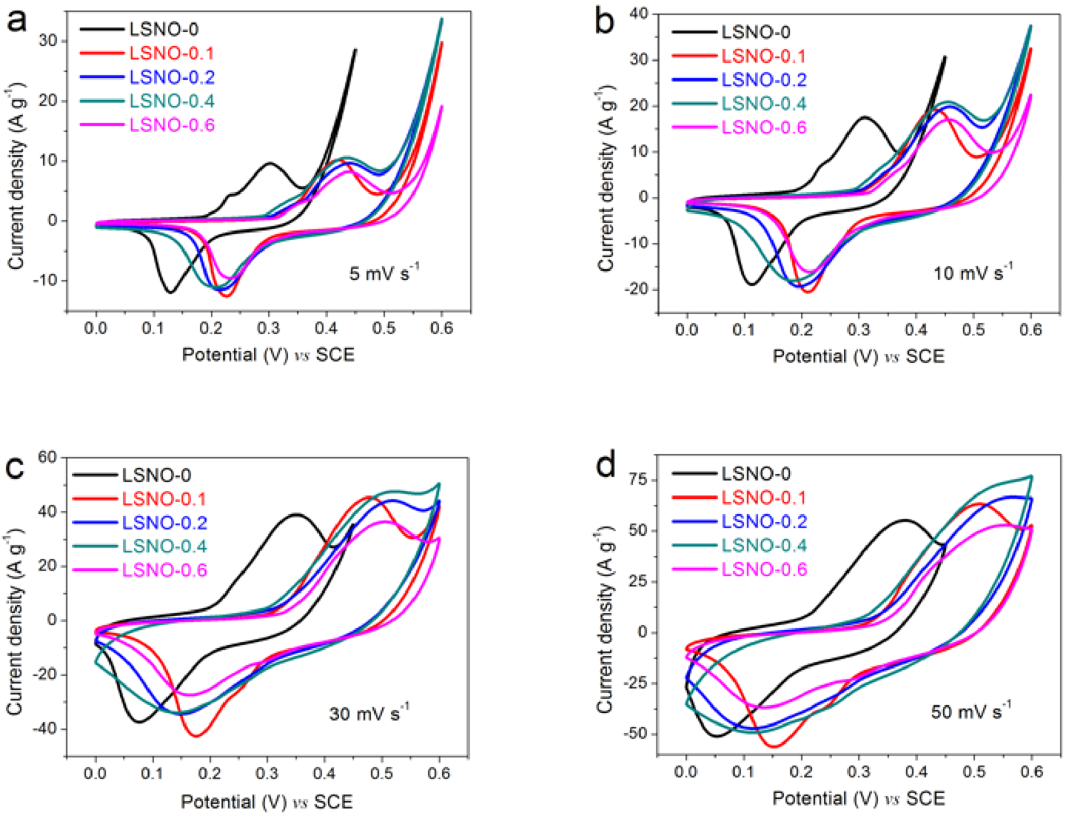

Figure S4. (**a–d**) Cyclic voltammetry (CV) curves of the LSNO-0, LSNO-0.1, LSNO-0.2 and LSNO-0.6 electrodes at different scan rates ranging from 5 to 50 mV s^{-1}.

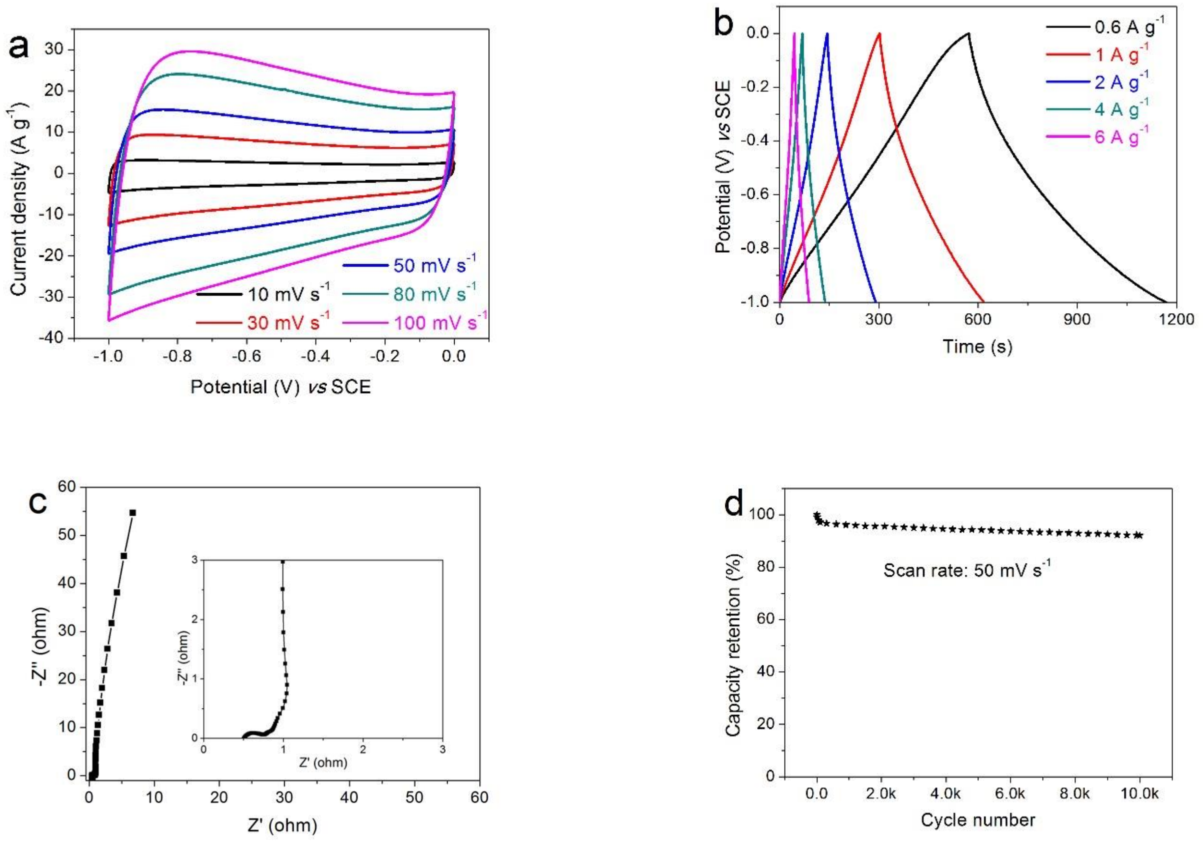

Figure S5. (**a**) CV curves, (**b**) GCD curves, (**c**) Nyqusit plots and (**d**) Cycling performance of the prepared activated carbon (AC) electrode in 6 M KOH electrolyte.

The electrochemical performance of the purchased AC electrode are measured by cyclic voltammetry (CV), galvanostatic charge/discharge (GCD) and electrochemical impedance spectroscopy (EIS) in a conventional three-electrode system with 6 M KOH aqueous solution as electrolyte. As illustrated in Figure S5a, all of the CV curves at various scanning rates present quasi-rectangular shapes, showing a typical characteristic of the relatively ideal electric double layer capacitance (EDLC). The GCD curves display an approximately symmetrical triangles (Figure S5b), which further explains that the capacity mainly produce from the adsorption and desorption of cations and anions on the surface of the electrode active material, and is a physical process. And a slightly bending curve existed in the charge curve of the current density of 0.6 A g^{-1} indicates the existence of a small amount of Faraday capacity, which is due to the redox reaction occurred in a small number of functional groups on the surface of AC materials at a small current. Furthermore, the AC electrode also exhibits a high capacity of 100.3 mAh g^{-1} at a current density of 0.6 A g^{-1}. The Nyqusit plots of AC electrode collected in the frequency ranging from 100 kHz to 0.01 Hz is displayed in Figure S5c. The approximately vertical line in the low frequency region is corresponded to a very small diffusion resistance, and a small semicircle radius in intermediate frequency region is corresponded to a small charge-transfer resistance, indicating an excellent electrical conductivity. The cycle stability of AC electrode shown in Figure S5d indicates an excellent cycling performance with 92.1% capacity retention over 10,000 cycles at a scan rate of 50 mV s^{-1}. These test results show that AC electrode is an ideal material with excellent electrochemical performance.

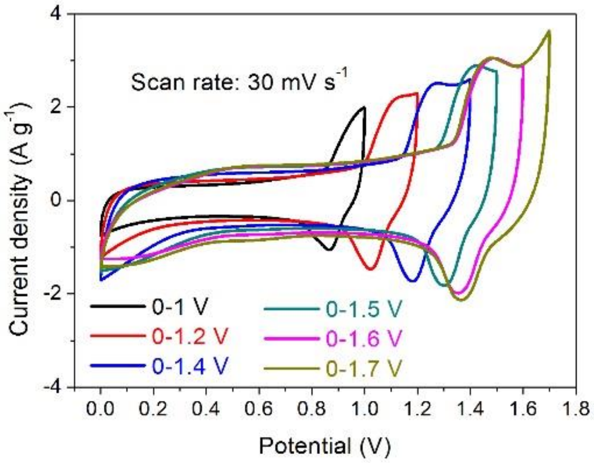

Figure S6. CV curves at different potential window ranging from 0–1 V to 0–1.7 V at a scan rate of 30 mV s^{-1}.

Table S1. Textural parameters of the LSNO-x samples.

Sample	S_{BET} (m^2 g^{-1})	Sm (m^2 g^{-1})	D_{DFT} (nm)	V (cm^3 g^{-1})	Vm/V(%)
LSNO-0	36	2	30.62	0.13	1.12%
LSNO-0.1	43	3	28.16	0.13	1.34%
LSNO-0.2	42	7	21.71	0.14	5.34%
LSNO-0.4	48	16	18.48	0.22	15.2%
LSNO-0.6	30	6	26.29	0.11	4.88%

Note: S_{BET} is the BET surface area; Sm is the t-Plot micropore area; D_{DFT} is the DFT desorption average pore diameter; V is the total pore volume; Vm/V is the percentage of the t-Plot micropore volume (Vm) to the total pore volume (V).

Table S2. Comparison of capacitive performance based on ABO_3-based electrode materials in the literatures.

Samples	*Sc* (F g^{-1})	Current density (Scan rate)	capacitance retention (cycle numbers)	Electrolyte	Ref.
$SrCo_{0.9}Nb_{0.1}O_{3-\delta}$	773.6	0.5 A g^{-1}	95.7% (3000)	KOH	[1]
Sr doped $LaNiO_3$ nanofibers	719	2 A g^{-1}	/	1 M Na_2SO_4	[2]
$LaNiO_3$	422	1 A g^{-1}	~83% (5000)	6 M KOH	[3]
$LaNiO_3$/NiO	9.5 mf cm^2	0.1V s^{-1}	97.2% (1000)	1 M Na_2SO_4	[4]
CeO_2 mixed $LaMnO_3$ nanocomposites	262	1 A g^{-1}	98% (2000)	1 M Na_2SO_4	[5]
$La_{0.85}Sr_{0.15}MnO_3$	102	1 A g^{-1}	/	1 M KOH	[6]
$La_{0.85}Sr_{0.15}MnO_3$	198	0.5 A g^{-1}	~77% (1000)	1 M KOH	[7]
$La_{0.6}Sr_{0.4}NiO_{3-\delta}$	115.88 mAh g^{-1} (851.32)	0.6 A g^{-1}	104.4% (16000)	6 M KOH	This work

References

1. L. Zhu, Y. Liu, C. Su, W. Zhou, M.L. Liu, Z.P. Shao, Perovskite $SrCo_{0.9}Nb_{0.1}O_{3-\delta}$ as an Anion-Intercalated Electrode Material for Supercapacitors with Ultrahigh Volumetric Energy Density, *Angew. Chem.* 2016, *55* 9576–9579.
2. Y. Cao, B.P. Lin, Y. Sun, H. Yang, X.Q. Zhang, Sr-doped Lanthanum Nickelate Nanofibers for High Energy Density Supercapacitors, *Electrochim. Acta* **2015**, *174*, 41–50.

3. T.Y. Shao, H.H. You, Z.J. Zhai, T.H. Liu, M. Li, L. Zhang, Hollow spherical $LaNiO_3$ supercapacitor electrode synthesized by a facile template-free method, *Mater. Lett.* **2017**, *201*, 122–124.
4. K. Liang, N. Wang, M. Zhou, Z.Y. Cao, T.L. Gu, Q. Zhang, X.Z. Tang, W.C. Hu, B.Q. Wei, Mesoporous $LaNiO_3$/NiO nanostructured thin films for high-performance supercapacitors, *J. Mater. Chem. A* **2013**, *1*, 9730–9736.
5. S. Nagamuthu, S. Vijayakumar, K.S. Ryu, Cerium oxide mixed $LaMnO_3$ nanoparticles as the negative electrode for aqueous asymmetric supercapacitor devices, *Mater. Chem. Phys.* **2017**, *199*, 543–551.
6. X.Q. Lang, H.Y. Mo, X.Y. Hu, H.W. Tian, Supercapacitor performance of perovskite $La_{1-x}Sr_xMnO_3$, *Dalton T.* **2017**, *46*, 13720–13730.
7. X.W. Wang, Q.Q. Zhu, X.E. Wang, H.C. Zhang, J.J. Zhang, L.F. Wang, Structural and electrochemical properties of $La_{0.85}Sr_{0.15}MnO_3$ powder as an electrode material for supercapacitor, *J. Alloy. Compd.* **2016**, *675*, 195–200.
